# Determinants of stadium attendance in Italian Serie A: New evidence based on fan expectations

**DOI:** 10.1371/journal.pone.0261419

**Published:** 2021-12-14

**Authors:** Francesco Addesa, Alexander John Bond

**Affiliations:** Sport Management Group, Carnegie School of Sport, Leeds Beckett University, Leeds, United Kingdom; Institute for Economic Forecasting, Romanian Academy, ROMANIA

## Abstract

This article aims to analyse the impact of the main determinants of match-day stadium attendance for seven seasons—2012–13 to 2018–19—of the Italian football Serie A. The main element of novelty is that the dataset is split into three sub-categories based on the pre-season fans’ expectations to verify whether the impact of attendance determinants varies depending on teams’ expected performance. Our results—based on Tobit model regressions—identify some significant differences across the three subsets. However, the difference that seems to be the most significant revealed a common preference of Italian fans towards higher quality opponents.

## Introduction

Sports economists have a long history of modelling demand at live sports events since seminal work [1–3]. Research especially soared in the early 2000s, perhaps due to the exponential commercial growth of professional sport. Generally, sports economists analyse stadium attendance following Borland and McDonald’s model, encompassing consumer preferences, economic factors, quality of viewing, sporting contest and supply capacity [4]. Among these categories of attendance determinants, Rottenberg’s [1] uncertainty of outcome hypothesis has garnered the most interest, suggesting that the more uncertain the outcome of a sporting event, the higher the interest will be [5–7].

However, empirical research investigating the uncertainty-of-outcome hypothesis provides inconsistent evidence: especially at match level, this hypothesis rarely holds in European football [8–14]. Since many leagues have a multi-prize structure leading to multiple sub-competitions, the fixture-specific uncertainty may not matter. However, the importance of that fixture within the league sub-competitions does. Therefore, the number of teams in contention for the different sub-competitions may drive match-level demand in European football rather than uncertainty-of-outcome, as proven by more recent empirical studies [12, 13, 15–19].

Another explanation of the limited evidence supporting the uncertainty-of-outcome hypothesis in European football is the reference-dependent preferences model [20], which bases fans’ decisions on prospect theory [21]. Here, demand is a function of individuals’ decision-making under uncertainty, where demand increases as the opponent becomes inferior (*loss aversion*) or superior (*David v Goliath effect* [10]): either way, demand increases as match certainty increases. Furthermore, prospect theory suggests that non-expected utility maximisation significantly influences aspects of decisions made under uncertainty [22]. Consequently, competing for a sub-competition within a league which is higher than expected should significantly increase demand in the context of competitive intensity. Likewise, competing for a sub-competition within a league which is lower than expected should decrease demand. Therefore, this article analyses Italian Serie A match-day attendance between 2012–13 to 2018–19 (seven seasons), split into three sub-categories based on the pre-season fans’ expectations. This approach follows previous work [16] and allows us to verify whether the impact of attendance determinants varies depending on teams’ expected performance.

## Materials and methods

The study uses a dataset of 2498 fixtures covering seven seasons of the Italian Serie A, from 2012/13 to the 2018/19 season. The league is made up of 20 teams playing each other in 38 game weeks within each season. Games were analysed starting from the third fixture of each season, as only one or two fixtures are not sufficient to differentiate among the different sporting prizes, which is instrumental to creating the variables capturing competitive intensity. Moreover, 22 games were excluded because of the following reasons; (i) not played, (ii) played behind closed doors, (iii) played in a city different from the one where the home team was based—due to stadium renovation works or other stadium-related issues, or, (iv) played with a reduced stadium capacity—lower than the number of season ticket holders. These were; 12 of Cagliari’s home games in 2012/13, 2 of Cagliari’s home games in 2013–14, Juventus vs Udinese and Napoli vs Verona in 2013–14, Palermo vs Atalanta in 2015–16, 2 of Crotone’s home games in 2016–17, and 2 of Atalanta’s home games and Inter vs Sassuolo in 2018–19.

The dataset was then divided into three subsets based on the expected final position for each club at the beginning of the season. Two types of pre-season expectations were considered, one based on Eurobet “ante-post” odds, the other on the teams’ overall payroll (Table 1). The first subset includes teams expected to end the season in the first seven positions, the second subset comprises teams expected to finish in the middle of the table (8^th^ to 13^th^ place), and the third includes those expected to finish at the bottom (14^th^ to 20^th^ place).

**Table 1:** Team subsets based on fan expectations.

Season	1–7			8–13			14–20		
	Predicted position	Eurobet ante-post	Team wages	Predicted position	Eurobet ante-post	Team wages	Predicted position	Eurobet ante-post	Team wages
2012–13	1	Juventus	Milan	8	Fiorentina	Sampdoria	14	Cagliari	Parma
	2	Milan	Juventus	9	Palermo	Genoa	15	Catania	Udinese
	3	Inter	Inter	10	Parma	Bologna	16	Sampdoria	Siena
	4	Roma	Roma	11	Bologna	Atalanta	17	Chievo	Catania
	5	Napoli	Lazio	12	Genoa	Palermo	18	Pescara	Cagliari
	6	Lazio	Napoli	13	Atalanta	Torino	19	Siena	Chievo
	7	Udinese	Fiorentina				20	Torino	Pescara
2013–14	1	Juventus	Juventus	8	Udinese	Genoa	14	Verona	Verona
	2	Milan	Milan	9	Genoa	Bologna	15	Bologna	Catania
	3	Napoli	Inter	10	Parma	Parma	16	Atalanta	Udinese
	4	Inter	Roma	11	Catania	Sampdoria	17	Cagliari	Sassuolo
	5	Fiorentina	Napoli	12	Sampdoria	Torino	18	Sassuolo	Chievo
	6	Roma	Lazio	13	Torino	Atalanta	19	Livorno	Cagliari
	7	Lazio	Fiorentina				20	Chievo	Livorno
2014–15	1	Juventus	Juventus	8	Udinese	Palermo	14	Cagliari	Atalanta
	2	Roma	Roma	9	Parma	Sampdoria	15	Chievo	Verona
	3	Napoli	Milan	10	Torino	Sassuolo	16	Palermo	Parma
	4	Inter	Inter	11	Atalanta	Genoa	17	Sampdoria	Chievo
	5	Fiorentina	Napoli	12	Genoa	Torino	18	Empoli	Cagliari
	6	Milan	Fiorentina	13	Verona	Udinese	19	Sassuolo	Cesena
	7	Lazio	Lazio				20	Cesena	Empoli
2015–16	1	Juventus	Juventus	8	Sampdoria	Sampdoria	14	Bologna	Palermo
	2	Roma	Roma	9	Torino	Bologna	15	Empoli	Torino
	3	Napoli	Milan	10	Genoa	Sassuolo	16	Atalanta	Verona
	4	Milan	Inter	11	Udinese	Genoa	17	Sassuolo	Chievo
	5	Inter	Napoli	12	Palermo	Atalanta	18	Chievo	Empoli
	6	Lazio	Lazio	13	Verona	Udinese	19	Frosinone	Carpi
	7	Fiorentina	Fiorentina				20	Carpi	Frosinone
2016–17	1	Juventus	Juventus	8	Sassuolo	Sampdoria	14	Chievo	Atalanta
	2	Roma	Inter	9	Torino	Bologna	15	Atalanta	Cagliari
	3	Napoli	Roma	10	Genoa	Torino	16	Cagliari	Palermo
	4	Inter	Milan	11	Sampdoria	Genoa	17	Bologna	Chievo
	5	Milan	Napoli	12	Empoli	Sassuolo	18	Palermo	Pescara
	6	Fiorentina	Lazio	13	Udinese	Udinese	19	Pescara	Empoli
	7	Lazio	Fiorentina				20	Crotone	Crotone
2017–18	1	Juventus	Juventus	8	Atalanta	Sampdoria	14	Chievo	Verona
	2	Napoli	Milan	9	Torino	Fiorentina	15	Bologna	Cagliari
	3	Milan	Roma	10	Sampdoria	Bologna	16	Genoa	Spal
	4	Roma	Inter	11	Sassuolo	Sassuolo	17	Verona	Udinese
	5	Inter	Napoli	12	Udinese	Atalanta	18	Benevento	Chievo
	6	Lazio	Lazio	13	Cagliari	Genoa	19	Spal	Benevento
	7	Fiorentina	Torino				20	Crotone	Crotone
2018–19	1	Juventus	Juventus	8	Fiorentina	Fiorentina	14	Bologna	Atalanta
	2	Napoli	Milan	9	Torino	Sampdoria	15	Cagliari	Udinese
	3	Inter	Inter	10	Sampdoria	Bologna	16	Chievo	Parma
	4	Roma	Roma	11	Genoa	Sassuolo	17	Empoli	Frosinone
	5	Milan	Napoli	12	Udinese	Cagliari	18	Parma	Chievo
	6	Lazio	Lazio	13	Sassuolo	Genoa	19	Spal	Spal
	7	Atalanta	Torino				20	Frosinone	Empoli

For each of the three groups, we estimated different specifications of a demand model, including variables capturing most of the determinants of attendance identified by [4] and inspired by other works investigating these determinants without differentiating the analysis against fans’ expectations [16, 17]:

$$ln\left({matchday\_tickets}_{ijt}\right)=\alpha X_{ijt}+\beta Z+\;\gamma S+\;e_{ijt}$$ (1)

Our dependant variable is the number of match-day tickets sold, excluding the season tickets—obtained from www.stadiapostcards.com. It is essential to exclude season-ticket holders for match-level analysis as they pre-purchase all fixtures regardless of the peculiar characteristics of a game [8]. *X*_*ijt*_ is a vector of independent variables, *Z* a vector of dummy variables, whereas *S* is a vector of dummies capturing season fixed effects, *α*, *β*, and *γ* are the associated coefficients, and *e*_*ijt*_ the disturbance term.

Tobit regressions [22] with individual cut-off points were estimated to account for the supply capacity constraint on attendance [4] and the consequential truncation of the number of match-day tickets sold at the upper boundary [23]. We used "available" tickets—measured as the difference between the stadium capacity and the number of season ticket sales—as the individual cut-off points. Consequently, 18 observations within the Tobit model were right-censored for the whole dataset. Regressions were performed using the *metobit* command in Stata 16 software [24].

The explanatory variables were aligned to [4]. To account for economic factors, we included a) the annual unemployment rate of the municipality where the game was played (*unemployment*); b) market size measured by the total number of supporters of the two teams across the whole Italian territory (*home_fans* and *away_fans*) as a result of a survey conducted by the specialised website www.tifosobilanciato.it; c) distance, in km, between the two cities of teams involved in the game (*distance*), proxying the travel cost for away fans and used in previous works [25, 26] and d) substitution effect by measuring the number of games scheduled at the same time and broadcast on TV (*substitutes*). It is worth mentioning that, in the seven seasons under investigation, all the Serie A matches were broadcast on Sky: therefore, the inclusion of a dummy variable capturing the “direct” substitute effect is redundant [4, 9, 10, 27–33].

To account for the quality of viewing factors, we included a) a set of integer (*temperature*, *humidity*) and dummy (*rain*, *storm*, *fog*, *snow*) variables for weather conditions; and b) a set of dummy variables for the timing of contest: *working_day*, indicating whether a match was scheduled on a weekday or not [17, 34, 35]; *sat_aft*, for matches scheduled at 3 pm on Saturdays; *sat_eve*, for matches scheduled at 6 pm on Saturdays; *sat_nig*, for matches with a 8.45 pm kick-off on Saturdays; *sun_eve*, for matches scheduled at 6 pm on Sundays; *sun_nig* and *sun_noon* for matches with a 8.45 pm and 12.30 pm kick-off on Sundays, respectively. The dummy capturing matches scheduled at 3 pm on Sundays was excluded from the model since it showed a Variance Inflation Factor (VIF) coefficient higher than 10, leading to the presence of strong collinearity in our estimates. Its high VIF can be explained by its strong correlation with the variable *substitutes*. Unsurprising, since the games with the higher number of substitutes are the traditional 3 pm Sunday kick-off. We decided to exclude a variable capturing the quality of stadium facilities since there has been limited stadium investment in Italian football. For example, only three new stadiums have been built in the last decade (Juventus Stadium in September 2011; Dacia Arena in January 2016 for Udinese; and Benito Stirpe Stadium, in September 2017, for Frosinone). Consequently, most stadiums are quite obsolete with minor repairs, making it difficult to identify an objective measure of the status of their facilities.

Finally, we included a set of variables capturing the characteristics of the sporting contest [4]. The count of matches in each season (*fixture*) was included in quadratic form to verify the existence of a non-linear relationship with the number of spectators, as first suggested in [11]. *home_rank* indicates the position in the standings of the home team before the game, and *home_wages* is a control variable capturing their relative wage—the ratio between the team’s payroll and the average seasonal payroll. The opponent’s quality is proxied by their position in the standings (*away_rank*) and their relative wage (*away_wages*). *home_promotion* and *away_promotion* are dummy variables aimed at capturing whether newly promoted teams attract more fans in home and away games. Whereas *goal_average* is the sum of the goal averages scored by home and away teams before the game, and *rivalry* is a dummy equal to 1 if the game involves two rival clubs and 0 otherwise. We considered the rivalries identified by Paliotto [36] and based not only on the clubs’ geographical location but also on the historical sensibility of fan groups [37].

The uncertainty of outcome variable (*outcome_uncertainty*) is calculated as the absolute difference between the home and the away team win probabilities [9], which is more sensitive to the actual gap between teams than draw probabilities [10] and is obtained from the *BET365* dataset. The two dummy variables *ncs_prize* and *pcs_prize*, measure any negative and positive change in the standing during the home team’s last two games, determine a change in the prize gained, and aim to capture the league standing effect [2]. Three variables were then used to account for competitive intensity [38–40], implying that demand is stimulated when more teams are in contention for multi-prize sub-competitions (qualification to playoffs or international competitions and promotion-relegation, among others). *top* indicates whether the home team was fighting for the title (1^st^ place), a direct entry to the Champions League (2^nd^ place until 2016/17; 2^nd^ to 4^th^ place in 2017–18 and 2018–19) or an entry to the Champions League qualifying round (3^rd^ place until 2016–17). *europa* indicates direct entry to the Europa League (4^th^ and 5^th^ place until 2016–17; 5^th^ and 6^th^ place in 2017–18–2018–19) or an entry to the Europa League qualifying round (6^th^ place until 2016–17; 7^th^ place in 2017–18–2018–19). *bottom* indicates the avoidance of the last three positions, determining relegation. As of 2017–18, the Italian league qualifies four teams directly to the UEFA Champions League group stage, following a reform in the admission rules to the competition and an improved UEFA ranking coefficient [41]. Moreover, if the Coppa Italia winner did not finish the season in the first five positions (six as of 2017–18), they would gain direct qualification to the Europa League group stage, so that the team finishing 5^th^ (6^th^ as of 2017–18) would gain an entry to the Europa League qualifying round rather than the team finishing 6^th^ (7^th^ as of 2017–18). This occurrence happened twice during the period under investigation, in 2012–13 and 2018–19. However, the Coppa Italia final is scheduled at the end of the season; therefore, it is reasonable to assume that, with the season underway, fans still look at the position in the standings as decisive for the Europa League qualification.

The three dummies capturing competitive intensity are functions of the point difference for the home team relating to the league prizes [12, 16, 17]. These are determined by taking into account three temporal horizons: the next match, the next two (*i*) and the next three matches (*ii*). For example, a team was considered in contention for a prize in the first temporal horizon if there was a gap of no more than 3 points. If the home team was in contention for more than one prize among Champions League and Europa League qualification within the same temporal horizon, only the highest prize was taken into account (1 for this prize, 0 for the other prizes). For example, if a team was 3 points ahead of the first team out of the Europa League zone and 2 points behind the Champions League zone, it was considered in contention for the Champions League qualification. In the two-match temporal horizon, if one match (e.g., 3 points) was sufficient for a higher prize (e.g., Europa League qualification) whereas two matches (e.g., 5 points) were required for a lower prize (e.g., relegation battle), we took into account the higher prize. In the three-match temporal horizon, if two matches (e.g., 6 points) were sufficient for a lower prize and three matches (e.g., 7 points) were required for a higher prize, we took into account the lower prize. When we estimated our model with the inclusion of the three-match temporal horizon competitive intensity, games were analysed starting from the fourth fixture of each season, which reduced our dataset from 2498 to 2428 games.

Table 2 below provides sources and descriptive statistics for all the variables for the whole dataset.

**Table 2:** Descriptive statistics and data sources.

Variable	Obs	Mean	Std. Dev.	Min	Max	Source
*matchday_tickets*	2,498	9136.50	9551.68	53	82442	www.stadiapostcards.com
*unemployment*	2,498	10	5	4.55	28.65	www.istat.it
*home_fans*	2,498	1167651	1842301	13508	7086915	www.tifosobilanciato.it
*away_fans*	2,498	1166977	1841198	13508	7086915	
*distance*	2,498	433.10	289.10	0.001	1300	www.viamichelin.com
*substitutes*	2,498	2.09	2.31	0	7	www.legaseriea.it
*temperature*	2,498	12.55	5.85	-3	28	www.ilmeteo.it
*humidity*	2,498	73.52	14.28	10	100	
*rain*	2,498	0.38	0.48	0	1	
*storm*	2,498	0.08	0.28	0	1	
*fog*	2,498	0.14	0.35	0	1	
*snow*	2,498	0.01	0.10	0	1	
*working_day*	2,498	0.17	0.37	0	1	www.legaseriea.it
*sat_aft*	2,498	0.05	0.21	0	1	
*sat_eve*	2,498	0.09	0.29	0	1	
*sat_nig*	2,498	0.09	0.29	0	1	
*sun_eve*	2,498	0.05	0.21	0	1	
*sun_nig*	2,498	0.11	0.31	0	1	
*sun_noon*	2,498	0.08	0.28	0	1	
*fixture*	2,498	20.49	10.36	3	38	
*home_rank*	2,498	10.21	5.75	1	20	
*away_rank*	2,498	10.06	5.74	1	20	
*home_wages*	2,498	46.73	37.93	8	219	www.gazzetta.it
*away_wages*	2,498	46.62	37.96	8	219	
*home_promotion*	2,498	0.15	0.36	0	1	www.legaseriea.it
*away_promotion*	2,498	0.15	0.36	0	1	
*goal_average*	2,498	2.69	0.72	0.4	6	
*rivalry*	2,498	0.09	0.29	0	1	
*outcome_uncertainty*	2,498	0.33	0.22	0	0.91	www.bet365.com
*ncs_prize*	2,498	0.09	0.29	0	1	www.legaseriea.it
*pcs_prize*	2,498	0.06	0.24	0	1	
*top*	2,498	0.22	0.42	0	1	
*europa*	2,498	0.16	0.37	0	1	
*bottom*	2,498	0.18	0.39	0	1	
*top_i*	2,498	0.34	0.47	0	1	
*euuropa_i*	2,498	0.16	0.37	0	1	
*bottom_i*	2,498	0.28	0.45	0	1	
*top_ii*	2,428	0.39	0.49	0	1	
*europa_ii*	2,428	0.15	0.36	0	1	
*bottom_ii*	2,428	0.34	0.47	0	1	

## Results and discussion

Our results are shown in Tables 3–10. Our regressions were performed using the whole set of explanatory variables. However, for a clearer presentation of the results, we have divided them into four groups based on Borland and MacDonald’s categorisation of the determinants of attendance [4]. Results seem relatively consistent regardless of the type of pre-season expectations used to create the three subsets. All the explanatory variables—except for ordinal (*fixture*, *home_rank* and *away_rank*) and dummy variables—are expressed in natural logs, and the estimated coefficients are interpreted as elasticities. Dummies capturing season fixed effects are not reported for the sake of brevity. Variance inflation factors (VIF)—calculated for the whole dataset—are significantly lower than 10 for our independent variables, proving the absence of strong collinearity (see S1 Appendix). Robust standard errors are estimated as the Breusch-Pagan test reveals the presence of heteroskedasticity.

**Table 3:** “Economic” determinants of attendance in Italian Serie A: Sub-groups based on BET 365 antepost.

	1–7			8–13			14–20		
	(1)	(2)	(3)	(1)	(2)	(3)	(1)	(2)	(3)
*unemployment*	0.774***	0.782***	0.795***	0.081	0.053	0.055	0.080	0.070	0.057
	(0.051)	(0.050)	(0.052)	(0.099)	(0.097)	(0.101)	(0.052)	(0.052)	(0.053)
*home_fans*	0.383***	0.386***	0.370***	0.229***	0.235***	0.234***	0.086***	0.097***	0.089***
	(0.043)	(0.044)	(0.044)	(0.034)	(0.034)	(0.035)	(0.023)	(0.023)	(0.024)
*away_fans*	0.017	0.013	0.014	0.065*	0.068*	0.072**	0.099***	0.096***	0.102***
	(0.023)	(0.023)	(0.024)	(0.035)	(0.035)	(0.036)	(0.033)	(0.033)	(0.033)
*distance*	-0.040***	-0.042***	-0.040***	-0.057***	-0.057***	-0.055***	-0.064***	-0.066***	-0.062***
	(0.009)	(0.009)	(0.010)	(0.015)	(0.015)	(0.015)	(0.014)	(0.014)	(0.015)
*substitutes*	-0.005	-0.005	-0.004	-0.017	-0.026	-0.032	-0.030	-0.034	-0.023
	(0.043)	(0.043)	(0.045)	(0.061)	(0.061)	(0.063)	(0.052)	(0.052)	(0.052)
*Sigma*	0.280***	0.277***	0.280***	0.408***	0.407***	0.406***	0.366***	0.363***	0.367***
	(0.031)	(0.030)	(0.030)	(0.028)	(0.027)	(0.028)	(0.026)	(0.026)	(0.027)
*Observations*	877	877	855	756	756	732	865	865	841

*Notes*. Robust standard errors in parentheses obtained using the robust or sandwich estimator of variance: p*<0.10, p**<0.05, p***<0.01.

No newly promoted team in the first two subsets.

**Table 4:** “Economic” determinants of attendance in Italian Serie A: Sub-groups based on team overall wages.

	1–7			8–13			14–20		
	(1)	(2)	(3)	(1)	(2)	(3)	(1)	(2)	(3)
*unemployment*	0.593***	0.612***	0.618***	-0.199**	-0.214**	-0.190**	0.190***	0.169***	0.158***
	(0.050)	(0.049)	(0.051)	(0.092)	(0.092)	(0.094)	(0.062)	(0.061)	(0.062)
*home_fans*	0.455***	0.456***	0.447***	0.149***	0.150***	0.142***	0.143***	0.156***	0.151***
	(0.042)	(0.042)	(0.043)	(0.023)	(0.023)	(0.024)	(0.032)	(0.032)	(0.033)
*away_fans*	0.014	0.009	0.010	0.072**	0.069**	0.067**	0.096***	0.097***	0.104***
	(0.023)	(0.022)	(0.024)	(0.032)	(0.032)	(0.032)	(0.035)	(0.036)	(0.036)
*distance*	-0.037***	-0.039***	-0.038***	-0.071***	-0.071***	-0.067***	-0.087***	-0.087***	-0.088***
	(0.009)	(0.009)	(0.009)	(0.013)	(0.014)	(0.014)	(0.017)	(0.016)	(0.018)
*substitutes*	-0.045	-0.047	-0.035	0.006	0.009	0.024	-0.043	-0.057	-0.051
	(0.042)	(0.042)	(0.043)	(0.054)	(0.054)	(0.056)	(0.055)	(0.055)	(0.056)
*Sigma*	0.259***	0.254***	0.258***	0.329***	0.328***	0.330***	0.430***	0.430***	0.429***
	(0.027)	(0.027)	(0.027)	(0.017)	(0.017)	(0.017)	(0.035)	(0.035)	(0.036)
*Observations*	879	879	856	756	756	734	863	863	838

*Notes*. Robust standard errors in parentheses obtained using the robust or sandwich estimator of variance: p*<0.10, p**<0.05, p***<0.01.

No newly promoted team in the first subset.

**Table 5:** “Quality of viewing” determinants of attendance in Italian Serie A: Sub-groups based on BET 365 antepost.

	1–7			8–13			14–20		
	(1)	(2)	(3)	(1)	(2)	(3)	(1)	(2)	(3)
*temperature*	0.212***	0.200***	0.219***	-0.076	-0.082	-0.089	0.031	0.035	0.028
	(0.070)	(0.067)	(0.070)	(0.095)	(0.097)	(0.096)	(0.120)	(0.123)	(0.117)
*humidity*	0.136	0.097	0.090	0.262**	0.250**	0.259**	0.136	0.124	0.116
	(0.098)	(0.093)	(0.095)	(0.109)	(0.111)	(0.110)	(0.135)	(0.138)	(0.138)
*rain*	-0.097**	-0.089**	-0.083*	-0.194***	-0.192***	-0.192***	-0.105**	-0.100**	-0.098*
	(0.043)	(0.043)	(0.043)	(0.056)	(0.055)	(0.056)	(0.049)	(0.050)	(0.051)
*storm*	0.032	0.038	0.061	-0.184	-0.183	-0.221*	0.049	0.057	0.077
	(0.068)	(0.067)	(0.069)	(0.115)	(0.116)	(0.124)	(0.083)	(0.082)	(0.089)
*fog*	0.078	0.076	0.099*	0.057	0.042	0.031	0.051	0.069	0.062
	(0.055)	(0.056)	(0.057)	(0.079)	(0.079)	(0.079)	(0.067)	(0.066)	(0.066)
*snow*	-0.078	-0.010	-0.010	0.319	0.307	0.320	-0.461	-0.463*	-0.524*
	(0.138)	(0.128)	(0.152)	(0.231)	(0.233)	(0.235)	(0.285)	(0.271)	(0.292)
*working_day*	-0.387***	-0.377***	-0.398***	-0.183***	-0.175***	-0.170***	-0.046	-0.045	-0.046
	(0.060)	(0.059)	(0.061)	(0.083)	(0.082)	(0.082)	(0.064)	(0.064)	(0.065)
*sat_aft*	0.005	0.028	0.006	0.122	0.140	0.145	0.016	-0.000	0.013
	(0.094)	(0.092)	(0.089)	(0.095)	(0.097)	(0.096)	(0.090)	(0.088)	(0.087)
*sat_eve*	-0.173**	-0.150*	-0.143*	-0.099	-0.077	0.076	0.098	0.099	0.109
	(0.079)	(0.079)	(0.080)	(0.102)	(0.102)	(0.103)	(0.090)	(0.090)	(0.091)
*sat_nig*	-0.093	-0.092	-0.082	0.080	0.054	0.040	-0.063	-0.102	-0.043
	(0.064)	(0.065)	(0.067)	(0.120)	(0.119)	(0.122)	(0.097)	(0.096)	(0.100)
*sun_eve*	-0.048	-0.053	-0.034	-0.048	-0.047	-0.057	0.138	0.148	0.169
	(0.078)	(0.080)	(0.082)	(0.125)	(0.122)	(0.131)	(0.104)	(0.103)	(0.105)
*sun_nig*	-0.224***	-0.218***	-0.207***	0.152	0.144	0.156	-0.049	-0.056	0.004
	(0.062)	(0.062)	(0.065)	(0.113)	(0.111)	(0.119)	(0.096)	(0.095)	(0.104)
*sun_noon*	-0.063	-0.032	-0.061	0.224**	0.206**	0.192*	0.057	0.049	0.067
	(0.095)	(0.097)	(0.099)	(0.103)	(0.101)	(0.104)	(0.095)	(0.094)	(0.097)
*Sigma*	0.280***	0.277***	0.280***	0.408***	0.407***	0.406***	0.366***	0.363***	0.367***
	(0.031)	(0.030)	(0.030)	(0.028)	(0.027)	(0.028)	(0.026)	(0.026)	(0.027)
*Observations*	877	877	855	756	756	732	865	865	841

*Notes*. Robust standard errors in parentheses obtained using the robust or sandwich estimator of variance: p*<0.10, p**<0.05, p***<0.01.

No newly promoted team in the first two subsets.

**Table 6:** “Quality of viewing” determinants of attendance in Italian Serie A: Sub-groups based on team overall wages.

	1–7			8–13			14–20		
	(1)	(2)	(3)	(1)	(2)	(3)	(1)	(2)	(3)
*temperature*	0.269***	0.248***	0.278***	-0.143**	-0.144**	-0.153**	0.134	0.144	0.128
	(0.064)	(0.062)	(0.064)	(0.072)	(0.073)	(0.073)	(0.126)	(0.130)	(0.128)
*humidity*	0.149	0.123	0.103	0.261***	0.279***	0.280***	-0.085	-0.112	-0.114
	(0.094)	(0.090)	(0.092)	(0.096)	(0.095)	(0.094)	(0.169)	(0.172)	(0.173)
*rain*	-0.119***	-0.111***	-0.108**	-0.182***	-0.179***	-0.167***	-0.043	-0.033	-0.039
	(0.043)	(0.042)	(0.042)	(0.051)	(0.050)	(0.051)	(0.054)	(0.054)	(0.055)
*storm*	0.020	0.032	0.043	-0.068	-0.059	-0.063	-0.017	-0.008	-0.013
	(0.066)	(0.065)	(0.068)	(0.086)	(0.088)	(0.090)	(0.106)	(0.105)	(0.116)
*fog*	0.010	0.006	0.029	0.286***	0.278***	0.271***	0.032	0.058	0.052
	(0.055)	(0.055)	(0.057)	(0.062)	(0.062)	(0.061)	(0.076)	(0.075)	(0.075)
*snow*	-0.083	-0.024	0.007	-0.254	-0.255	-0.310	0.271	0.204	0.228
	(0.164)	(0.161)	(0.175)	(0.262)	(0.268)	(0.258)	(0.215)	(0.195)	(0.206)
*working_day*	-0.340***	-0.327***	-0.353***	-0.191***	-0.188***	-0.181**	-0.036	-0.036	-0.030
	(0.060)	(0.059)	(0.061)	(0.071)	(0.071)	(0.073)	(0.072)	(0.071)	(0.071)
*sat_aft*	0.009	0.035	0.019	0.004	0.005	0.003	0.095	0.082	0.105
	(0.089)	(0.090)	(0.086)	(0.100)	(0.101)	(0.101)	(0.82)	(0.082)	(0.081)
*sat_eve*	-0.123	-0.093	-0.086	0.135	0.125	0.144	0.097	0.098	0.110
	(0.083)	(0.080)	(0.081)	(0.087)	(0.086)	(0.088)	(0.094)	(0.093)	(0.094)
*sat_nig*	-0.069	-0.070	-0.059	0.058	0.051	0.059	-0.023	-0.051	-0.019
	(0.064)	(0.065)	(0.066)	(0.097)	(0.098)	(0.101)	(0.120)	(0.119)	(0.121)
*sun_eve*	0.001	-0.010	0.011	-0.117	-0.118	-0.103	0.118	0.120	0.157
	(0.077)	(0.080)	(0.082)	(0.132)	(0.133)	(0.139)	(0.094)	(0.095)	(0.097)
*sun_nig*	-0.196***	-0.185***	-0.179***	0.023	0.017	0.028	0.068	0.035	0.113
	(0.060)	(0.060)	(0.063)	(0.096)	(0.097)	(0.106)	(0.117)	(0.115)	(0.123)
*sun_oon*	-0.001	0.016	0.014	0.145	0.144	0.151	-0.009	-0.037	-0.041
	(0.076)	(0.077)	(0.079)	(0.094)	(0.093)	(0.094)	(0.106)	(0.105)	(0.108)
*Sigma*	0.259***	0.254***	0.258***	0.329***	0.328***	0.330***	0.430***	0.430***	0.429***
	(0.027)	(0.027)	(0.027)	(0.017)	(0.017)	(0.017)	(0.035)	(0.035)	(0.036)
*Observations*	879	879	856	756	756	734	863	863	838

*Notes*. Robust standard errors in parentheses obtained using the robust or sandwich estimator of variance: p*<0.10, p**<0.05, p***<0.01.

No newly promoted team in the first subset.

**Table 7:** “Game characteristics” determinants of attendance in Italian Serie A: Sub-groups based on BET 365 antepost.

	1–7			8–13			14–20		
	(1)	(2)	(3)	(1)	(2)	(3)	(1)	(2)	(3)
*fixture*	-0.004	-0.013	-0.013	-0.033**	-0.039***	-0.056***	-0.019	-0.017	-0.025
	(0.012)	(0.012)	(0.013)	(0.017)	(0.017)	(0.018)	(0.019)	(0.018)	(0.019)
*fixture2*	0.000	0.001**	0.001*	0.001***	0.001***	0.002***	0.001	0.001	0.001
	(0.000)	(0.000)	(0.000)	(0.000)	(0.000)	(0.000)	(0.000)	(0.000)	(0.000)
*home_rank*	-0.012	-0.014	-0.013	-0.015*	-0.12	-0.018*	-0.010	-0.003	0.001
	(0.009)	(0.009)	(0.009)	(0.009)	(0.010)	(0.010)	(0.008)	(0.008)	(0.008)
*away_rank*	-0.003	-0.002	-0.004	-0.027***	-0.027***	-0.024***	0.007	0.007	0.010
	(0.005)	(0.005)	(0.006)	(0.007)	(0.007)	(0.008)	(0.007)	(0.007)	(0.007)
*home_wages*	-0.185*	-0.202**	-0.162	-0.076	-0.067	-0.035	0.678**	0.679***	0.679***
	(0.098)	(0.099)	(0.104)	(0.153)	(0.153)	(0.154)	(0.096)	(0.096)	(0.099)
*away_wages*	0.269***	0.286***	0.242***	0.169*	0.168*	0.178**	0.334***	0.341***	0.332***
	(0.060)	(0.060)	(0.061)	(0.087)	(0.087)	(0.090)	(0.075)	(0.075)	(0.077)
*home_promotion*							0.043	0.047	0.036
							(0.044)	(0.044)	(0.045)
*away_promotion*	0.225***	0.228***	0.227***	0.212***	0.231***	0.230***	0.123*	0.123*	0.116
	(0.054)	(0.054)	(0.054)	(0.075)	(0.074)	(0.074)	(0.073)	(0.071)	(0.075)
*goal_average*	-0.140	-0.229	-0.166	-0.064	-0.102	-0.086	0.200	0.188	0.298
	(0.160)	(0.159)	(0.161)	(0.166)	(0.165)	(0.186)	(0.168)	(0.167)	(0.200)
*rivalry*	-0.068	-0.065	-0.088	0.335***	0.335***	0.316***	0.170**	0.150*	0.171**
	(0.065)	(0.065)	(0.067)	(0.117)	(0.121)	(0.123)	(0.078)	(0.077)	(0.081)
*Sigma*	0.280***	0.277***	0.280***	0.408***	0.407***	0.406***	0.366***	0.363***	0.367***
	(0.031)	(0.030)	(0.030)	(0.028)	(0.027)	(0.028)	(0.026)	(0.026)	(0.027)
*Observations*	877	877	855	756	756	732	865	865	841

*Notes*. Robust standard errors in parentheses obtained using the robust or sandwich estimator of variance: p*<0.10, p**<0.05, p***<0.01.

No newly promoted team in the first two subsets.

**Table 8:** “Game characteristics” determinants of attendance in Italian Serie A: Sub-groups based on team overall wages.

	1–7			8–13			14–20		
	(1)	(2)	(3)	(1)	(2)	(3)	(1)	(2)	(3)
*fixture*	0.003	-0.007	-0.006	-0.051***	-0.055***	-0.065***	-0.005	-0.004	-0.020
	(0.011)	(0.011)	(0.012)	(0.015)	(0.014)	(0.015)	(0.019)	(0.020)	(0.021)
*fixture2*	0.000	0.000*	0.000	0.001***	0.002***	0.002***	0.000	0.000	0.001
	(0.000)	(0.000)	(0.000)	(0.000)	(0.000)	(0.000)	(0.000)	(0.000)	(0.001)
*home_rank*	-0.015*	-0.018**	-0.012	0.004	0.007	0.011	-0.027***	-0.022**	-0.022**
	(0.008)	(0.008)	(0.008)	(0.009)	(0.009)	(0.009)	(0.008)	(0.008)	(0.009)
*away_rank*	-0.002	-0.001	-0.003	-0.008	-0.009	-0.009	-0.005	-0.005	-0.001
	(0.005)	(0.005)	(0.005)	(0.007)	(0.007)	(0.007)	(0.007)	(0.008)	(0.008)
*home_wages*	-0.551***	-0.556***	-0.530***	-0.877***	-0.885***	-0.750***	0.815***	0.787***	0.769***
	(0.099)	(0.097)	(0.101)	(0.250)	(0.248)	(0.251)	(0.157)	(0.158)	(0.161)
*away_wages*	0.281***	0.291***	0.250***	0.199**	0.213***	0.219***	0.358***	0.355***	0.352***
	(0.059)	(0.058)	(0.060)	(0.079)	(0.079)	(0.080)	(0.079)	(0.079)	(0.081)
*home_promotion*				0.181**	0.205**	0.161*	0.210***	0.209***	0.206***
				(0.082)	(0.083)	(0.084)	(0.057)	(0.057)	(0.059)
*away_promotion*	0.208***	0.208***	0.214***	0.165**	0.185***	0.190***	0.208***	0.211***	0.207***
	(0.053)	(0.052)	(0.053)	(0.071)	(0.069)	(0.070)	(0.079)	(0.077)	(0.081)
*goal_average*	0.119	0.042	0.096	0.353**	0.294*	0.276	0.021	0.011	0.095
	(0.160)	(0.158)	(0.160)	(0.165)	(0.166)	(0.187)	(0.158)	(0.159)	(0.192)
*rivalry*	-0.039	-0.031	-0.054	0.309***	0.301***	0.311***	-0.011	-0.032	-0.022
	(0.063)	(0.062)	(0.065)	(0.087)	(0.090)	(0.091)	(0.102)	(0.102)	(0.103)
*Sigma*	0.259***	0.254***	0.258***	0.329***	0.328***	0.330***	0.430***	0.430***	0.429***
	(0.027)	(0.027)	(0.027)	(0.017)	(0.017)	(0.017)	(0.035)	(0.035)	(0.036)
*Observations*	879	879	856	756	756	734	863	863	838

*Notes*. Robust standard errors in parentheses obtained using the robust or sandwich estimator of variance: p*<0.10, p**<0.05, p***<0.01.

No newly promoted team in the first subset.

**Table 9:** “Uncertainty-of-outcome” determinants of attendance in Italian Serie A: Sub-groups based on BET 365 antepost.

	1–7			8–13			14–20		
	(1)	(2)	(3)	(1)	(2)	(3)	(1)	(2)	(3)
*outcome_uncertainty*	-0.708***	-0.703***	-0.805***	0.612***	0.619***	0.620***	0.830***	0.815***	0.827***
	(0.212)	(0.216)	(0.217)	(0.197)	(0.196)	(0.200)	(0.167)	(0.165)	(0.170)
*ncs_prize*	-0.015	0.037	0.054	-0.086	-0.084	-0.082	0.066	0.062	0.077
	(0.054)	(0.051)	(0.054)	(0.110)	(0.105)	(0.110)	(0.082)	(0.078)	(0.082)
*pcs_prize*	0.093	0.143**	0.150**	-0.037	-0.009	-0.031	0.012	0.004	0.006
	(0.064)	(0.063)	(0.063)	(0.103)	(0.097)	(0.099)	(0.114)	(0.113)	(0.121)
*top*	0.317***			0.147			-0.085		
	(0.055)			(0.120)			(0.115)		
*europa*	0.172***			-0.025			0.009		
	(0.052)			(0.085)			(0.086)		
*bottom*	0.049			0.205***			0.107*		
	(0.148)			(0.077)			(0.060)		
*top_i*		0.419***			0.214**			0.139	
		(0.061)			(0.103)			(0.091)	
*europa_i*		0.223***			0.007			0.221***	
		(0.073)			(0.093)			(0.084)	
*bottom_i*		0.161			0.193**			0.187***	
		(0.127)			(0.076)			(0.063)	
*top_ii*			0.464***			0.110			0.197**
			(0.082)			(0.115)			(0.096)
*europa_ii*			0.268***			0.006			0.325***
			(0.102)			(0.107)			(0.099)
*bottom_ii*			0.277*			0.215**			0.228***
			(0.142)			(0.096)			(0.069)
*Sigma*	0.280***	0.277***	0.280***	0.408***	0.407***	0.406***	0.366***	0.363***	0.367***
	(0.031)	(0.030)	(0.030)	(0.028)	(0.027)	(0.028)	(0.026)	(0.026)	(0.027)
*Observations*	877	877	855	756	756	732	865	865	841

*Notes*. Robust standard errors in parentheses obtained using the robust or sandwich estimator of variance: p*<0.10, p**<0.05, p***<0.01.

No newly promoted team in the first two subsets.

**Table 10:** “Uncertainty-of-outcome” determinants of attendance in Italian Serie A: Sub-groups based on team overall wages.

	1–7			8–13			14–20		
	(1)	(2)	(3)	(1)	(2)	(3)	(1)	(2)	(3)
*outcome_uncertainty*	-0.425***	-0.443***	-0.537***	0.739***	0.746***	0.708***	0.486***	0.500***	0.504***
	(0.204)	(0.206)	(0.210)	(0.196)	(0.196)	(0.201)	(0.175)	(0.174)	(0.177)
*ncs_prize*	-0.037	0.007	0.017	-0.065	-0.050	-0.019	0.022	0.014	0.025
	(0.051)	(0.049)	(0.052)	(0.086)	(0.084)	(0.089)	(0.110)	(0.108)	(0.115)
*pcs_prize*	0.127**	0.170***	0.170***	-0.013	0.006	0.008	-0.021	-0.036	-0.046
	(0.064)	(0.063)	(0.062)	(0.088)	(0.085)	(0.088)	(0.119)	(0.115)	(0.122)
*top*	0.307***			0.077			-0.138		
	(0.053)			(0.104)			(0.140)		
*europa*	0.157***			0.065			-0.115		
	(0.052)			(0.075)			(0.101)		
*bottom*	0.095			0.154**			0.198***		
	(0.182)			(0.079)			(0.063)		
*top_i*		0.440***			0.137			0.120	
		(0.059)			(0.092)			(0.112)	
*europa _i*		0.264***			0.156**			0.045	
		(0.069)			(0.078)			(0.112)	
*bottom_i*		0.243*			0.167**			0.253***	
		(0.137)			(0.074)			(0.069)	
*top_ii*			0.448***			0.013			0.163
			(0.078)			(0.096)			(0.120)
*europa_ii*			0.203**			0.201**			0.148
			(0.096)			(0.084)			(0.128)
*bottom_ii*			0.281**			0.065			0.335***
			(0.140)			(0.083)			(0.079)
*Sigma*	0.259***	0.254***	0.258***	0.329***	0.328***	0.330***	0.430***	0.430***	0.429***
	(0.027)	(0.027)	(0.027)	(0.017)	(0.017)	(0.017)	(0.035)	(0.035)	(0.036)
*Observations*	879	879	856	756	756	734	863	863	838

*Notes*. Robust standard errors in parentheses obtained using the robust or sandwich estimator of variance: p*<0.10, p**<0.05, p***<0.01.

No newly promoted team in the first subset.

### Economic factors

There are similarities and differences among the three subsets regarding the economic factors of attendance, as shown in Tables 3 and 4. Distance impacts all the clubs’ attendance regardless of the fan expectations. Less travel time is a good predictor of attendance since increased travel time often represents increased opportunity cost associated with attending a game for the supporters of the away team [27, 42–46]. The home team’s market size is another factor that shows the same (positive impact) attendance in all three subsets: a larger fanbase corresponds to a higher demand for tickets. However, the unemployment rate of the city hosting the home team is significant in both tables only for the first subset. The positive coefficient of this variable is not surprising if we consider that previous research showed that the relationship between unemployment and attendance is ambiguous [47, 48]. Since the first subset includes all the clubs from the four most populous Italian cities (Rome, Milan, Naples and Turin), the positive coefficient of the unemployment variable may indicate that football attendance is considered a social outlet for unemployed people in the metropolitan areas. This conclusion could be indeed extended to high unemployment areas, if we consider that in Table 4 the coefficient is significant and positive also for the third subset, but negative for the second, and the average unemployment rates across the period considered indicate that first and third subsets show values (11.14% and 10.76% respectively) significantly higher than the second subset (8.93%).

Conversely, the away team’s market size was significant only for the other two subsets. Therefore, when clubs are not expecting to fight for top finishing positions, the opposing team’s market size is significantly positive. Since those clubs with larger market sizes often resemble those competing for top prizes, three reasons may explain this. Firstly, the loss-acceptance from prospect theory [21] and the possibility of an upset against a team competing for a top prize. Secondly, fans gain utility from watching higher level performances from the opposition. Thirdly, teams with larger market sizes have fan bases spanning across the country. Therefore, demand increases as they play in other areas outside of their locality.

The number of games scheduled simultaneously and broadcast on TV does not have any impact on any of the three subsets. Therefore, there is no evidence of a substitution effect with other games. This finding shows that even single ticket buyers in the Italian Serie A demonstrate a certain degree of loyalty since the concurrence of other—potentially more appealing—games does not influence the decision to attend a game regardless of the expected team performance.

### Quality of viewing

Tables 5 and 6 show the coefficients of the variables linked to the quality of viewing in line with [4]. When considering weather conditions, the only variable that consistently affects attendance is *rain*, with the expected negative sign [5, 28, 49–51]. In contrast, the *temperature* only impacts positively the top 7 clubs’ fans in both tables and negatively the second subset in Table 6. This last result may depend on the fact that most games played with warmer weather conditions are scheduled towards the end of the season, when teams expected to finish between the eighth and the thirteenth position could see an increase in meaningless games if they are not in contention for any prize.

Interestingly, kick-off time does not have a consistent impact on attendance to games scheduled for weekends across the three subsets. We can only observe a clear aversion of the top 7 clubs’ fans to the Sunday night games in both tables and Saturday evening games in Table 5, where there is also a positive coefficient for the games scheduled at noon on Sundays in the second subset. In contrast, games scheduled for working days attract fewer spectators to home games for teams expected to finish in the first 13 positions. In the analysis of these variables, we find more similarities than differences across the three subsets, as both weather conditions and scheduling of contests do not generally show robust evidence of impact on attendance.

### Sporting contest: Game characteristics

Within the game characteristics category of factors (Tables 7 and 8), *fixture* consistently negatively impacts attendance for teams expected to finish between eighth and the thirteenth positions. Such a finding may reflect the increase in meaningless games if they are not in contention for any prize as the season progresses, consistent with our findings regarding the negative impact of *temperature* on the same subset. There is no consistent evidence that the home team position in the standing positively influences attendance, as *the home_rank* coefficient is significant only for the second subset in Table 7, for the other subsets—especially the third one—in Table 8. This may suggest that a better position in the standings could have a positive impact especially on supporters of the teams expected to fight to avoid the relegation. Regarding *away_rank*, its coefficient is significant only for the second subset in Table 7. Therefore, only supporters of the teams expected to finish between the eighth and the thirteenth position seem attracted by opponents that are having a better season performance. However, if we consider the away teams’ payroll, there was a consistent significant positive impact across all the sub-groups, especially in the third one.

Consequently, the model demonstrates—consistently with previous studies [14, 17, 31, 34, 35]–that the opponents’ quality matters to all fans, but more so to fans of lower-performing clubs. Interestingly the *home_wages* coefficients show a negative sign for the first and second subsets and a positive sign for the third. This finding possibly reflects how a home team’s talent stimulates season-ticket sales rather than match tickets, especially for fans who are used to higher quality players, unlike fans of teams expected to fight to avoid relegation.

The enthusiasm of newly promoted teams’ fans—potentially stimulating demand—is not consistently reflected by an increase in the home games. The *home_promotion* coefficients are significant only in Table 8—but in the away games’ attendance, indicating that the promotion effect impacts more on loyal fans, who are most likely to buy season tickets and follow their team to away games. The fact that away promotion coefficients are higher in the first subset may again indicate the relevance of the opponents’ quality: that said, we would need specific data on the number of tickets sold to away team supporters to obtain definitive conclusions.

A higher expected number of goals scored in a game does not impact attendance across the three subsets. When teams face a rival opponent instead, ticket sales increase for clubs expecting to finish after the seventh place (sub-groups 2 and 3), implying lower table clubs’ fans are more attracted by historical rivalry than big clubs’ fans. We also conducted further tests, replacing *rivalry* with a dummy variable accounting for matches between clubs in the same region and a dummy variable accounting for matches between clubs in the same city. We first kept *distance* among the explanatory variables, then took it off because it may capture part of the variability as in regional or local derbies; the distance between the two cities is limited. Including *distance* among the regressors, coefficients for both regional derbies and city derbies are still not significant for the first subset. Conversely, without *distance*, they become significant for positions 1–7, yet still smaller than positions 8–20. Further confirming the conclusion that rivalry is more critical to lower-performing clubs’ fans than top-performing clubs’ fans within the Italian Serie A. Results of these further tests are available upon request.

### Sporting contest: Uncertainty-of-outcome

The most interesting result of this research is undoubtedly related to the much-debated uncertainty-of-outcome hypothesis (Tables 9 and 10). There is a significant difference between the first subset and the other two. The models for the first subset (positions 1–7) show negative *outcomeuncertainty* coefficients. However, models for the other two subsets (positions 8–20) show the opposite. Consequently, the uncertainty-of-outcome hypothesis holds for the high performing clubs, suggesting their fans prefer a balanced game. In contrast, all other clubs experience higher demand when one of the two teams shows a clear competitive edge. Consequently, this demonstrates how clubs expecting to finish below seventh place demonstrate reference-dependent preferences, exhibiting traits of loss-aversion and loss-acceptance.

The fact that the uncertainty-of-outcome hypothesis is verified for the top 7 clubs but not the other two subsets may be simply a confirmation that the quality of opponents matters to all fans [17, 34, 35]. Comparing the top 7 teams’ expected with their actual performance, regardless of predictions based on betting odds or team wages, for five out of the seven seasons considered only one team did not manage to finish in the top 7, and only two teams for the other two seasons (2013–14 and 2014–15). Considering that the difference between home and away team win probabilities heavily depends on the quality and current performance of the teams involved, arguably the most balanced games for the top 7 teams are those played against teams in the same subset. Our evidence confirms this by a significantly lower average absolute difference between the two teams’ winning probabilities when both teams involved in a match belonged to the first subset (0.13 when based on "ante-post" betting odds, 0.23 when based on team wages) than when a team in the first subset hosted one in the second (0.53 for both grouping methods) or the third (0.61 and 0.60 respectively).

Fans supporting clubs expected to finish in the middle of the table are attracted by games where the absolute difference between the two teams’ winning probabilities is higher. Therefore, this is consistent with a convex relation between match-day tickets sold and home win probabilities emerging from an attendance model with reference-dependent preferences and loss-aversion [19]. The analysis of the average absolute difference between the two teams’ winning probabilities shows that the most unbalanced games were those where a team in the second subset hosted a team in the third (0.30 for both grouping methods). In these games the odds were—on average—in favour of the home teams, as the average non-absolute difference is equal to 0.30 for the first grouping method and 0.20 for the second. Consequently, this confirms loss-aversion characteristics for fans of teams expected to finish in the middle of the table as they are more attracted by games where their favourite team has higher winning probabilities. Games against teams in the first subset are—as expected—more unbalanced (average absolute difference equal to 0.25 for the first grouping method, 0.24 for the second) than games against teams in the same subset (0.21 and 0.19, respectively). The odds—on average—logically favour the away teams from the first subset (average non-absolute difference equal to -0.22 for both methods) and the home teams when playing games against teams in the same subset (0.18 for the first grouping method, 0.17 for the second). Therefore, regardless of whether home fans enjoy the possibility to admire or upset stronger opponents, quality still matters more than uncertainty-of-outcome for fans of clubs in the second subgroup.

In the third subset, comprising teams with the lowest overall quality (the average team wage is the lowest regardless of grouping method, see S2 Appendix), we also verify a higher preference for unbalanced games. The analysis of the average absolute difference between the two teams’ winning probabilities indicates that games where teams expected to fight to avoid the relegation hosted teams in the first subset show higher average values (0.35 for the first grouping method and 0.36 for the second) than the other games (0.19 and 0.20 respectively). If we consider the average non-absolute difference, we also find that the differential in favour of the home teams is higher when playing against teams in the same subset (0.17 for both grouping methods) than when playing against teams in the second subset (0.04 for the first grouping method and 0.06 for the second). This seems consistent with the above-mentioned attendance model with reference-dependent preferences [20].

Therefore, if we consider the quality of the opponents, the positive impact of uncertainty-of-outcome in the first subset is not necessarily in contradiction with the negative impact shown in the other two subsets. A higher opponents’ quality corresponds to a higher uncertainty of outcome for the top 7 clubs and a lower uncertainty of outcome for the others. Under this consideration, it indicates that opponent quality matters to all fans regardless of expected performance—a notion also demonstrated by the above-mentioned positive significance of away team wages coefficients for all the subsets.

Finally, further differences emerge when analysing the league standing effect, with the first subset (positions 1–7) sensitive to positive changes in the current position leading to contention for a better prize and the competitive intensity variables. Not surprisingly, clubs in the first subset see increased match-day ticket demand when fighting for the league title, Champions League qualification or Europa League qualification, with a clear preference for the first two. In contrast, *bottom* shows a significant coefficient only when the three-game horizon is taken into account. A situation that is more likely to occur in the early season when a top team may have had a bad start but is still not very distant from better prizes either. Europa League qualification seems to be an attractive prize for fans of clubs in subgroup 2 (positions 8–13), but only in Table 10, whereas demand consistently increases when they are fighting relegation—which, based on the pre-season expectations, would be a considerable underperformance. This is similar for teams expected to finish in the last seven positions, as coefficients for the *bottom* are significant in all the specifications, showing increased ticket sales for games when league survival is in doubt. Arguably, this demonstrates it could be financially more profitable—concerning the match-day revenue—to be in a relegation battle than secure in a mid-table position. In Table 9, we can also see that—not surprisingly—fighting for better prizes, a situation that is again more likely to occur in the early season, positively impacts attendance in the last subset. These results shed new light on previous work on European football, where the analysis was conducted without differentiating teams based on fan expectations [12, 16, 17] and the league standing effect turned out to be mostly insignificant, whereas being in contention for sporting prizes generally showed a positive impact on attendance.

## Conclusions

This article investigates the main determinants of attendance for the Italian Serie A. The large dataset enables us to create—for the first time—three subsets to explore potential similarities and differences in the behaviour of people supporting clubs expected to have different seasonal performances. This allows a more accurate consideration of the effects of fan expectations on attendance compared to Bond & Addesa [18], where they were simply embedded in the model for the whole dataset.

While we find some similarities across the three subsets, especially concerning weather conditions, scheduling and number of TV substitutes, a significant difference between the first subset and the other two seems to emerge when considering the sporting contest characteristics. Fans supporting teams expected to finish in the first seven positions seem to be particularly attracted by more balanced games, those supporting the other teams by more unbalanced games. A more in-depth analysis shows how these two results are not necessarily in contradiction, as this may indicate a general preference of Italian fans towards higher quality opponents. This notion is further supported by the positive significance of the variables capturing the quality of away teams for all the subsets. That said, higher home win probabilities are also a decisive factor in the second and third subsets, which seems consistent with the attendance model with reference-dependent preferences.

Regarding competitive intensity, Europa League qualification still has a certain appeal to top 7 clubs’ fans, perhaps representing a consolation prize, and for fans of clubs in the second subset. Surprisingly, fans of teams expected to finish between 8^th^ and 13^th^ positions are more attracted by games where their team is fighting to avoid relegation, which—less surprisingly—is also the most appealing prize for fans of teams expected to be in contention for it. The positive coefficients of *rivalry* only in the second and third subsets suggest that winning against rival teams may be considered an additional "prize" for mid-table and small clubs.

While our analysis presents significant differences in fan behaviour when accounting for the sub-groups of fans, based on their expectations, the main difference turned out to reveal a common preference of Italian fans towards higher quality opponents. Future research may try to replicate this study for other countries/leagues to verify whether this fan preference would also emerge in other contexts.

## Supporting information

S1 Appendix VIF statistics. This table shows the VIF coefficients of the variables included in the regression model.
(DOCX)

S2 Appendix Average team wage per subset. This table shows the average team wage for each of the three subsets in each season considered.
(DOCX)

S1 File Stata regressions and tests. This log file shows all the regressions and tests conducted on Stata.
(LOG)

S2 File Stata VIF. This log file shows how the VIF coefficients were obtained on Stata.
(LOG)
