# Supplementary material for: Determinants of stadium attendance in Italian Serie A: New evidence based on fan expectations
Source: PLoS One. 2021 Dec 14;16(12):e0261419. doi: 10.1371/journal.pone.0261419 (PMC8670676; doi:10.1371/journal.pone.0261419)
Supplement: S1 Appendix — This table shows the VIF coefficients of the variables included in the regression model. (DOCX) [file pone.0261419.s001.docx]

**Appendix A. VIF statistics**

|  | **VIF** | | |
| --- | --- | --- | --- |
| **Variable** | (1) | (2) | (3) |
| *unemployment* | 1.40 | 1.40 | 1.42 |
| *home_fans* | 5.59 | 5.59 | 5.55 |
| *away_fans* | 5.32 | 5.33 | 5.32 |
| *distance* | 1.41 | 1.41 | 1.42 |
| *substitutes* | 1.63 | 1.63 | 1.63 |
| *temperature* | 1.36 | 1.34 | 1.38 |
| *humidity* | 1.60 | 1.60 | 1.60 |
| *rain* | 1.41 | 1.41 | 1.42 |
| *storm* | 1.20 | 1.20 | 1.20 |
| *fog* | 1.32 | 1.32 | 1.32 |
| *snow* | 1.05 | 1.05 | 1.05 |
| *working_day* | 1.29 | 1.29 | 1.29 |
| *sat_aft* | 1.09 | 1.09 | 1.09 |
| *sat_eve* | 1.37 | 1.36 | 1.36 |
| *sat_nig* | 1.70 | 1.71 | 1.70 |
| *sun_eve* | 1.22 | 1.22 | 1.22 |
| *sun_nig* | 1.47 | 1.47 | 1.46 |
| *sun_noon* | 1.36 | 1.36 | 1.36 |
| *fixture* | 1.41 | 1.45 | 1.22 |
| *home_rank* | 3.87 | 4.42 | 4.45 |
| *away_rank* | 2.79 | 2.79 | 2.79 |
| *home_wages* | 6.87 | 6.91 | 6.92 |
| *away_wages* | 6.50 | 6.51 | 6.51 |
| *home_promotion* | 1.22 | 1.22 | 1.22 |
| *away_promotion* | 1.24 | 1.24 | 1.24 |
| *goal_average* | 2.79 | 2.80 | 2.79 |
| *rivalry* | 1.29 | 1.29 | 1.29 |
| *outcome_uncertainty* | 1.56 | 1.55 | 1.55 |
| *ncs_prize* | 1.15 | 1.09 | 1.08 |
| *pcs_prize* | 1.11 | 1.07 | 1.07 |
| *top* | 2.40 |  |  |
| *europa* | 1.52 |  |  |
| *bottom* | 1.58 |  |  |
| *top_i* |  | 3.35 |  |
| *europa_i* |  | 1.80 |  |
| *bottom_i* |  | 2.19 |  |
| *top_ii* |  |  | 3.41 |
| *europa_ii* |  |  | 1.92 |
| *bottom_ii* |  |  | 2.76 |
| *Mean* | 2.12 | 2.19 | 2.21 |
