# Supplementary material for: Determinants of stadium attendance in Italian Serie A: New evidence based on fan expectations
Source: PLoS One. 2021 Dec 14;16(12):e0261419. doi: 10.1371/journal.pone.0261419 (PMC8670676; doi:10.1371/journal.pone.0261419)
Supplement: S2 Appendix — This table shows the average team wage for each of the three subsets in each season considered. (DOCX) [file pone.0261419.s002.docx]

**Appendix B. Average team wage per subset**

|  | **(1-7)** | | **(8-13)** | | **(14-20)** | |
| --- | --- | --- | --- | --- | --- | --- |
| **Season** | **Eurobet ante-post** | **Team wages** | **Eurobet ante-post** | **Team wages** | **Eurobet ante-post** | **Team wages** |
| 2012-13 | 81.51 | 84.03 | 27.40 | 26.03 | 18.76 | 17.41 |
| 2013-14 | 86.30 | 86.30 | 26.72 | 28.70 | 21.09 | 19.39 |
| 2014-15 | 80.16 | 80.16 | 24.23 | 27.73 | 20.36 | 17.36 |
| 2015-16 | 86.29 | 86.29 | 24.67 | 26.33 | 18.57 | 17.14 |
| 2016-17 | 87.29 | 87.29 | 28.08 | 31.17 | 21.07 | 18.43 |
| 2017-18 | 90.29 | 91.71 | 30.00 | 30.50 | 20.43 | 18.57 |
| 2018-19 | 108.86 | 111.14 | 33.50 | 32.50 | 23.71 | 22.29 |
